# Web-Based Ultrasonic Nomogram Predicts Preoperative Central Lymph Node Metastasis of cN0 Papillary Thyroid Microcarcinoma

**DOI:** 10.3389/fendo.2021.734900

**Published:** 2021-09-07

**Authors:** Chunwang Huang, Shuzhen Cong, Shiyao Shang, Manli Wang, Huan Zheng, Suqing Wu, Xiuyan An, Zhaoqiu Liang, Bo Zhang

**Affiliations:** ^1^Department of Ultrasound, Guangdong Provincial People’s Hospital, Guangdong Academy of Medical Sciences, Guangzhou, China; ^2^The Second School of Clinical Medicine, Southern Medical University, Guangzhou, China; ^3^Department of Ultrasonic Imaging, Xiangya Hospital, Central South University, Changsha, China

**Keywords:** ultrasound, papillary thyroid microcarcinoma, clinically node-negative, central lymph node metastasis, nomogram, web-based

## Abstract

**Background:**

Many clinicians are facing the dilemma about whether they should apply the active surveillance (AS) strategy for managing Clinically Node-negative (cN0) PTMC patients in daily clinical practice. This research plans to construct a dynamic nomogram based on network, connected with ultrasound characteristics and clinical data, to predict the risk of central lymph node metastasis (CLNM) in cN0 PTMC patients before surgery.

**Methods:**

A retrospective analysis of 659 patients with cN0 PTMC who had underwent thyroid surgery and central compartment neck dissection. Patients were randomly (2:1) divided into the development cohort (439 patients) and validation cohort (220 patients). The group least absolute shrinkage and selection operator (Group Lasso) regression method was used to select the ultrasonic features for CLNM prediction in the development cohort. These features and clinical data were screened by the multivariable regression analysis, and the CLNM prediction model and web-based calculator were established. Receiver operating characteristic, calibration curve, Clinical impact curve and decision curve analysis (DCA) were used to weigh the performance of the prediction model in the validation set.

**Results:**

Multivariable regression analysis showed that age, tumor size, multifocality, the number of contact surface, and real-time elastography were risk factors that could predict CLNM. The area under the curve of the prediction model in the development and validation sets were 0.78 and 0.77, respectively, with good discrimination and calibration. A web-based dynamic calculator was built. DCA proved that the prediction model had excellent net benefits and clinical practicability.

**Conclusions:**

The web-based dynamic nomogram incorporating US and clinical features was able to forecast the risk of preoperative CLNM in cN0 PTMC patients, and has good predictive performance. As a new observational indicator, NCS can provide additional predictive information.

## Introduction

The increase in the occurrence of thyroid cancer is mainly due to the increase in the incidence of papillary thyroid microcarcinoma (PTMC). Hence, PTMC dominates in the diagnosis and treatment of thyroid cancer. Since most tumors are not clinically obvious or palpable, most PTMCs are diagnosed by high-resolution ultrasound (US) or US-guided fine-needle aspiration. With these techniques becoming more common, an increasing number of cases were diagnosed as PTMC (1), leading to over-diagnosis and over-treatment.

In the recent years, the active surveillance (AS) strategy for low-risk PTMC has been gradually adopted worldwide to avoid over-treatment. However, AS is not suitable for all PTMC cases. Among them, central lymph node metastasis (CLNM) is the predominant influencing component. CLNM is more general in PTMC patients and the occurrence can be as high as 64.1% (2). PTMC patients with CLNM usually require surgery. For Clinically Node-negative (cN0) PTMC, a growing number of physicians tend to apply AS. However, many clinicians are facing the dilemma about whether they should apply this management strategy in daily clinical practice (3). Despite the low probability of CLNM in cN0 PTMC, if metastasis occurs during AS, patients will have to undergo a wider range of treatments that may lead to more complications (4). Additionally, it is important to consider patient-specific psychological factors. Studies show that only 25% of patients choose AS when the cytological diagnosis is PTC (< 15 mm), while more patients choose surgery (5). Therefore, examination of cervical lymph nodes, particularly the central compartment lymph nodes, is essential for determining the surgical scope of PTMC thyroidectomy, for screening candidates suitable for AS, and for following disease progression during AS.

US is the primary method to evaluate TNM staging of PTMC before surgery and AS, which is helpful for diagnosis and evaluation of PTMC that may relapse (6). The occurrence of lateral lymph node metastasis (LLNM) was lower in PTMC than in CLNM, and US showed higher diagnostic accuracy for LLNM (7–9). Due to obesity (10), the interference from the thyroid, trachea, and other adjoining organs, the sensitivity of US in recognizing CLNM is unsatisfactory (11). Hence, to forecast CLNM in cN0 PTMC patients is extremely challenging, but also of high significance. More accurate prediction of the probability to develop CLNM will increase the willingness of clinicians and patients to opt for AS, when appropriate. Even if patients choose surgery out of fear or anxiety, it can also be used as a reference for the decision of performing central compartment neck dissection (CCND).

In the studies involving cN0 PTMC patients, the independent predictors of CLNM were not consistent (12–16). In addition, prediction of CLNM in cN0 PTMC patients is mostly centered on the postoperative period. Prediction is achieved by incorporating clinical data and pathological results (12, 13), or by combining ultrasonic features and clinical-pathological risk factors (14–16). Studies also proved that the ultrasonic predictors of cN0 PTMC can predict CLNM (14–16). However, preoperative forecast of CLNM US-based nomogram in cN0 PTMC patients and genuine visualization has not been investigated.

The purpose of this study was to advance and validate a US-based dynamic nomogram to foresee the hazard of CLNM in cN0 PTMC patients.

## Materials and Methods

### Patients

**Figure 1:**
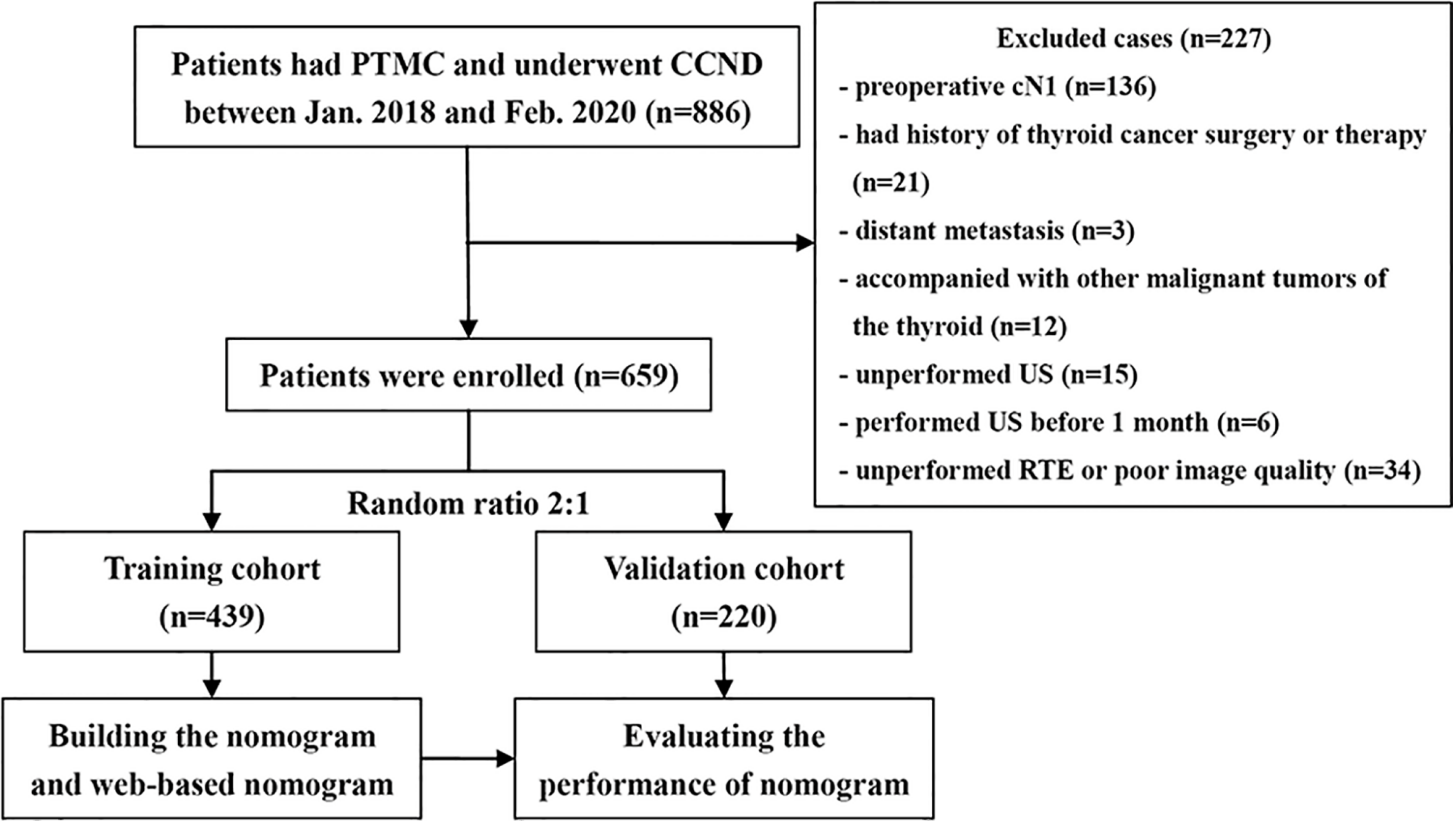
Flowchart of enrolled cN0 papillary thyroid microcarcinoma patients.

This research employed a single-center retrospective design, and all participated patients were anonymized. Hence, this research was permitted by the research ethics committee and the informed consent requirement was waved. Medical records from January 2018 to February 2020 were retrieved from our institution database. This research listed 659 patients totally with cN0 PTMC who met the demands. **Figure 1** shows the process of patient enrollment. According to the random allocation (2:1), the study cohort was divided into the development cohort consisting of 439 patients (100 men and 339 women, average age: 42.31 ± 9.06 years) and the validation cohort consisting of 220 patients (52 men, 168 women, average age: 44.00 ± 8.90 years).

Inclusion criteria: (1) patients who underwent thyroidectomy and CCND for the first time and were diagnosed with PTMC with histopathology; (2) status of CLNM were confirmed by pathology; (3) preoperative imaging diagnosis was negative for CLNM; (4) patients who had undergone US examination in our department one month before surgery; (5) the tumors underwent real-time elastography (RTE); (6) there were complete medical data.

Exclusion criteria: (1) patients who had thyroid carcinoma surgery or treatment before; (2) patients with other thyroid malignant tumors; (3) patients with distant metastasis or malignant tumors in other organs; (4) patients with few tumor foci or lymph node (LN) metastases, and pathological diagnosis of PTMC or CLNM metastasis could not be confirmed; (5) thyroid ablation was performed before surgery; (6) incomplete US image information, or poor image quality.

### US and RTE Images Analysis

During the study, all ultrasound examinations were performed by color Doppler diagnostic tools (Hitachi, Ltd., Tokyo, Japan), with high frequency of 6-13 MHz linear array probe. All patients with thyroid nodules experienced preoperative US examination, and data were stored for follow-up analysis.

Two independent radiologists with 11 and 16 years of thyroid imaging experience retrospectively analyzed these ultrasound pictures. In cases that the radiologists had different opinions, the third experts who have over 20 years of experience was involved in the discussion and a final decision was made. These three radiologists were not aware of patient’s medical data, and surgical and pathological results.

The sonographic features of the tumor under ultrasound were observed and recorded, including tumor size, tumor position, multifocality, bilateral tumor (0 represents unilateral multifocality, while 1 suggests bilateral multifocality), echogenicity, calcification patterns (Mode 0 represents no calcification, mode 1 represents microcalcification, and mode 2 represents coarse calcification. When coarse calcification and micro calcification coexisted in the lesion, it was classified as mode 1), number of contact surface (NCS, including anterior capsule, posterior capsule, upper pole, lower pole, medial and lateral, was evaluated based on US images. Capsule contact or involvement was defined as more than 25% of the tumor surface in contact or adjacent to the thyroid capsule on US images, or when the continuity of thyroid capsule echo around the tumor was interrupted. 0 represents that the tumor was not in contact with the capsule. 1 suggests that the tumor was in contact with one side of the capsule, while 2 represents that the tumor contacted ≥ two sides of the capsule), composition, shape, margin, taller-than-wide, chronic lymphocytic thyroiditis (CLT), internal color Doppler vascularity (The evaluation criteria were based on Adler grade classification (17), which represents semi quantitative classification), and cervical lymph node status (Suspicious US Features included calcification, cystic degeneration, increased cortical echo and/or rich or irregular blood flow in lymph nodes). When more than one suspicious thyroid malignancies were found, the relevant data of the largest suspicious tumor would be analyzed and recorded.

Routine US examination was followed by RTE, performed by the same radiologist. The same US machine and probe were used for elastography of thyroid nodules detected by conventional US. The elasticity score (ES) for each nodule would be assigned different points (0–4-point scale) according to Asteria et al. (18) standard. The values of RTE were: 0 for tumor ES 0-2; 1 for ES 3; 2 for ES 4.

### Statistical Analysis

All tests are two-sided tests, *P <*0.05, the difference is statistically significant. Continuous variables used Wilcoxon-Mann-Whitney U or test t-test. Pearson χ^2^ test or Fisher’s exact test was employed for categorical variables. Categorical variables used Fisher’s exact test or Pearson χ^2^ test. As there were many multi-categorical variables, the group least absolute shrinkage and selection operator (Group lasso) method using 10-fold cross-validation was applied to choose the most predictive US features in the development dataset. Multivariable logistic regression analysis was made on the above US risk factors and clinical data, and the risk predictors and regression coefficients were obtained, and the nomogram was generated. In the development and verification data set, the receiver operating characteristic (ROC) curve and calibration curve are derived to assess the performance of the web-based nomogram. In the validation set, decision curve analysis (DCA) and clinical impact curve were applied to assess the feasibility and application value of the optimization model in clinical practice (19). Using the “Dynnom” and “shiny” software packages of the R language, a web-based probability calculator is constructed.

SPSS 26.0 software (IBM Corp., Armonk, NY, USA) and R software (Rstudio version 1.3.1073, Rstudio Inc., Boston, MA, USA) were used for statistical analysis.

## Results

### US and Clinical Features

**Table 1** concludes the US features and clinical characteristics of the patients in the development and validation set. The positive rates of CLNM in the development and validation sets were 36.4% and 35% individually. Hence, the prevalence of CLNM in the two datasets had no significant difference (*P* =0.715). In addition, there were no significant differences in sex and age between the CLNM positive and the negative group, indicating the applicability of the development and validation data sets.

**Table 1:** Clinical and US features of patients in the development and validation cohorts.

Characteristic	Development cohort (n = 439)			Validation cohort (n = 220)		
	CLNM(-) (n = 279)	CLNM(+) (n = 160)	*P* value	CLNM(-) (n = 143)	CLNM(+) (n = 77)	*P* value
Age (mean ± SD, range, years)	43.47 ± 9.04(20-73)	40.29 ± 9.06 (19-69)	0.004	44.60 ± 8.92 (23-69)	42.91 ± 8.95 (17-66)	0.261
Sex (n, %)			0.024			0.024
Female	225 (80.6)	114 (71.3)		116 (81.1)	52 (67.5)	
Male	54 (19.4)	46 (28.7)		27 (18.9)	25 (32.5)	
Tumor Size(cm)	0.7 ± 0.15 (0.3-1.0)	0.76 ± 0.15 (0.3-1.0)	<0.001	0.7 ± 0.16 (0.3-1.0)	0.78 ± 0.16 (0.4-1.0)	0.002
Tumor Position (n, %)			0.357			0.014
left lobe	127 (45.5)	66 (41.3)		60 (42.0)	33 (42.9)	
right lobe	142 (50.9)	84 (52.5)		82 (57.3)	38 (49.3)	
isthmus & conical lobe	10 (3.6)	10 (6.2)		1 (0.7)	6 (7.8)	
Multifocality (n, %)			0.002			0.003
No	238 (85.3)	117 (73.1)		120 (83.9)	51 (66.2)	
Yes	41 (14.7)	43 (26.9)		23 (16.1)	26 (33.8)	
Bilateral Tumors (n, %)			0.001			<0.001
No	256 (91.8)	130 (81.3)		127 (88.8)	53 (68.8)	
Yes	23 (8.2)	30 (18.7)		16 (11.2)	24 (31.2)	
Very hypoechoic/hypoechoic (n, %)			0.213			0.103
No	27 (9.7)	10 (6.3)		12 (8.4)	12 (15.6)	
Yes	252 (90.3)	150 (93.7)		131 (91.6)	65 (84.4)	
Calcification (n, %)			0.012			0.167
0	111 (39.9)	43 (26.9)		54 (37.8)	21 (27.3)	
1	155 (55.5)	112 (70.0)		82 (57.3)	54 (70.1)	
2	13 (4.6)	5 (3.1)		7 (4.9)	2 (2.6)	
NCS (n, %)			<0.001			<0.001
0	180 (64.5)	73 (45.6)		95 (66.4)	31 (40.3)	
1	92 (33.0)	65 (40.6)		44 (30.8)	41 (53.2)	
≥2	7 (2.5)	22 (13.8)		4 (2.8)	5 (6.5)	
Composition (n, %)			NA			0.462
Cystic	0 (0)	0 (0)		1 (0.7)	0 (0)	
Solid	279 (100)	160 (100)		142 (99.3)	77 (100)	
Shape (n, %)			0.442			0.34
Regular	15 (5.4)	6 (3.8)		5 (3.5)	1 (1.3)	
Irregular	264 (94.6)	154 (96.2)		138 (96.5)	76 (98.7)	
Taller Than Wide (n, %)			0.914			0.525
≤1	57 (20.4)	32 (20.0)		23 (16.1)	15 (19.5)	
>1	222 (79.6)	128 (80.0)		120 (83.9)	62 (80.5)	
Margin (n, %)			0.929			0.734
Well-defined	10 (3.6)	6 (3.8)		7 (4.9)	3 (3.9)	
Ill-defined	269 (96.4)	154 (96.2)		136 (95.1)	74 (96.1)	
CLT (n, %)			0.314			0.245
Absence	184 (65.9)	113 (70.6)		95 (66.4)	57 (74.0)	
Presence	95 (34.1)	47 (29.4)		48 (33.6)	20 (26.0)	
Internal Vascularity (n, %)			0.148			0.196
0	51 (18.3)	17 (10.7)		35 (24.5)	11 (14.3)	
1	166 (59.5)	98 (61.2)		71 (49.6)	42 (54.5)	
2	29 (10.4)	21 (13.1)		19 (13.3)	16 (20.8)	
3	33 (11.8)	24 (15.0)		18 (12.6)	8 (10.4)	
RTE (n, %)			<0.001			<0.001
0	48 (17.2)	7 (4.4)		23 (16.1)	4 (5.2)	
1	214 (76.7)	94 (58.7)		106 (74.1)	43 (55.8)	
2	17 (6.1)	59 (36.9)		14 (9.8)	30 (39.0)	

NCS, Number of Contact Surface; CLT, chronic lymphocytic thyroiditis; RTE, real-time elastography.

### Construction of the Predictive Model and Nomogram

**Figure 2:**
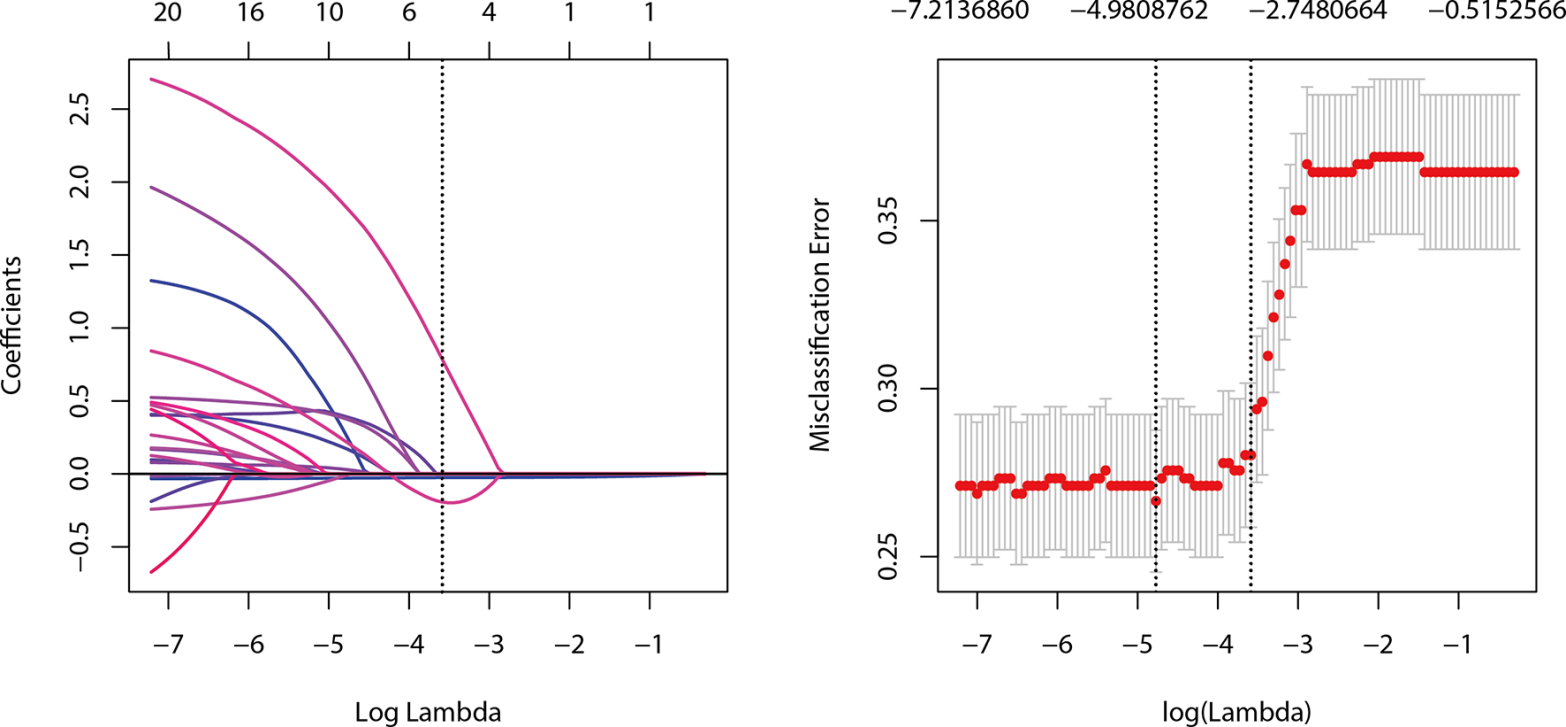
Selection of ultrasound features using the Group LASSO regression in the development dataset.

**Figure 3:**
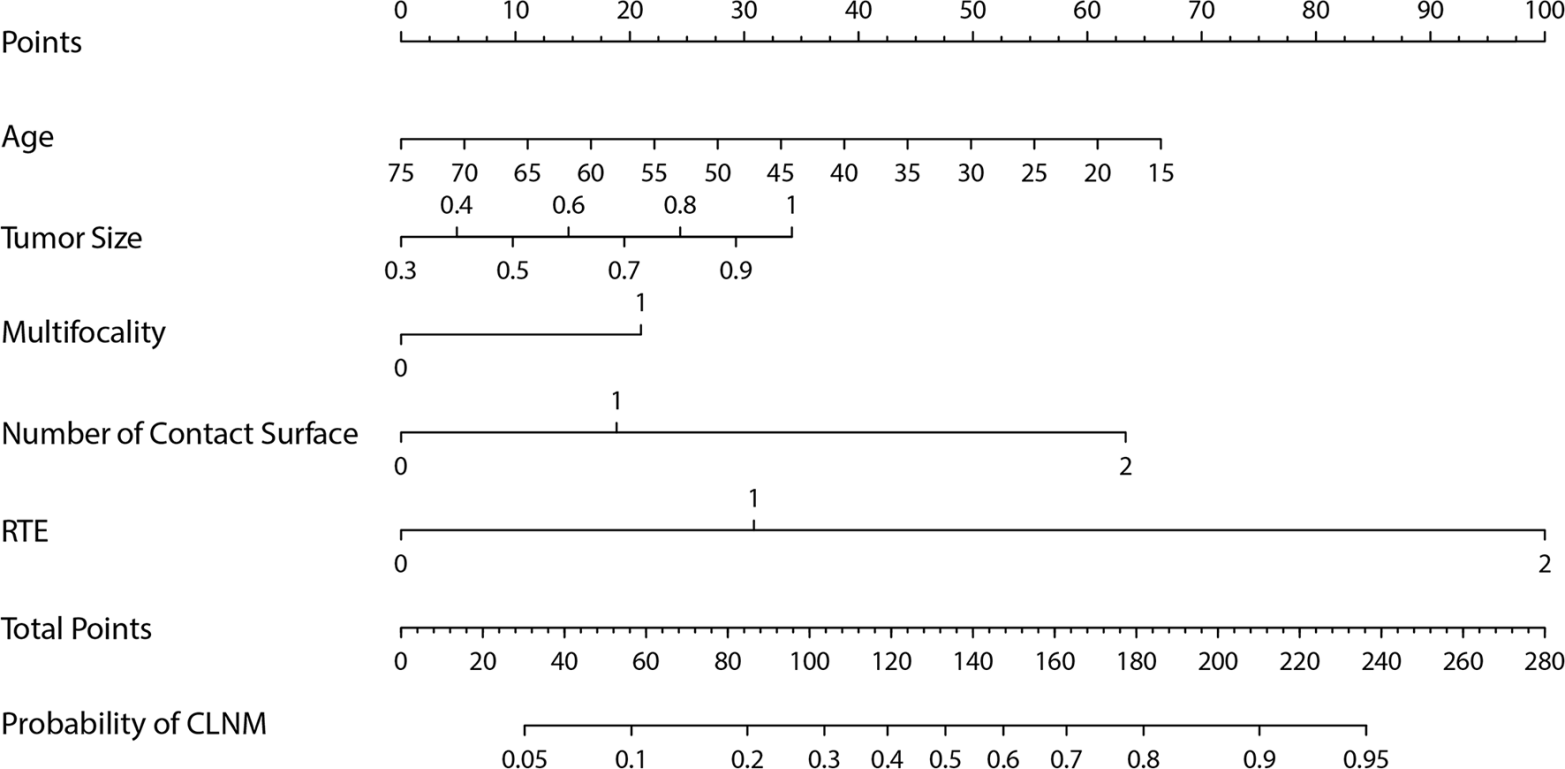
The nomogram for predicting the central lymph node metastasis probability in cN0 papillary thyroid microcarcinoma patients.

The Group Lasso was used to select the non-zero coefficient features of the prediction model in the development dataset (**Figure 2**). The 14 US features were reduced to four potential risk prediction factors: tumor size, multifocality, NCS, and RTE. Multivariable regression analysis presented that age was also a risk predictor in the prediction model (**Table 2**). The above five risk predictors were used to build a predictive model and plot a nomogram (**Figure 3**).

**Table 2:** Prediction factors and regression coefficients of the prediction model.

	Coefficient	OR (95% CI)	P value
(Intercept)	-1.854	0.157 (0.035-0.651)	0.012
Age	-0.032	0.969 (0.949-0.989)	0.003
Tumor Size	1.395	4.035 (1.137-14.659)	0.032
Multifocality			
0		1	
1	0.599	1.821 (1.052-3.148)	0.032
Number of Contact Surface			
0		1	
1	0.539	1.713 (1.072-2.745)	0.024
2	1.811	6.116 (2.402-17.131)	<0.001
RTE			
0		1	
1	0.882	2.415 (1.063-6.295)	0.049
2	2.858	17.434 (6.697-51.202)	<0.001

RTE, real-time elastography.

### Performance of US-Based Nomogram

**Figure 4:**
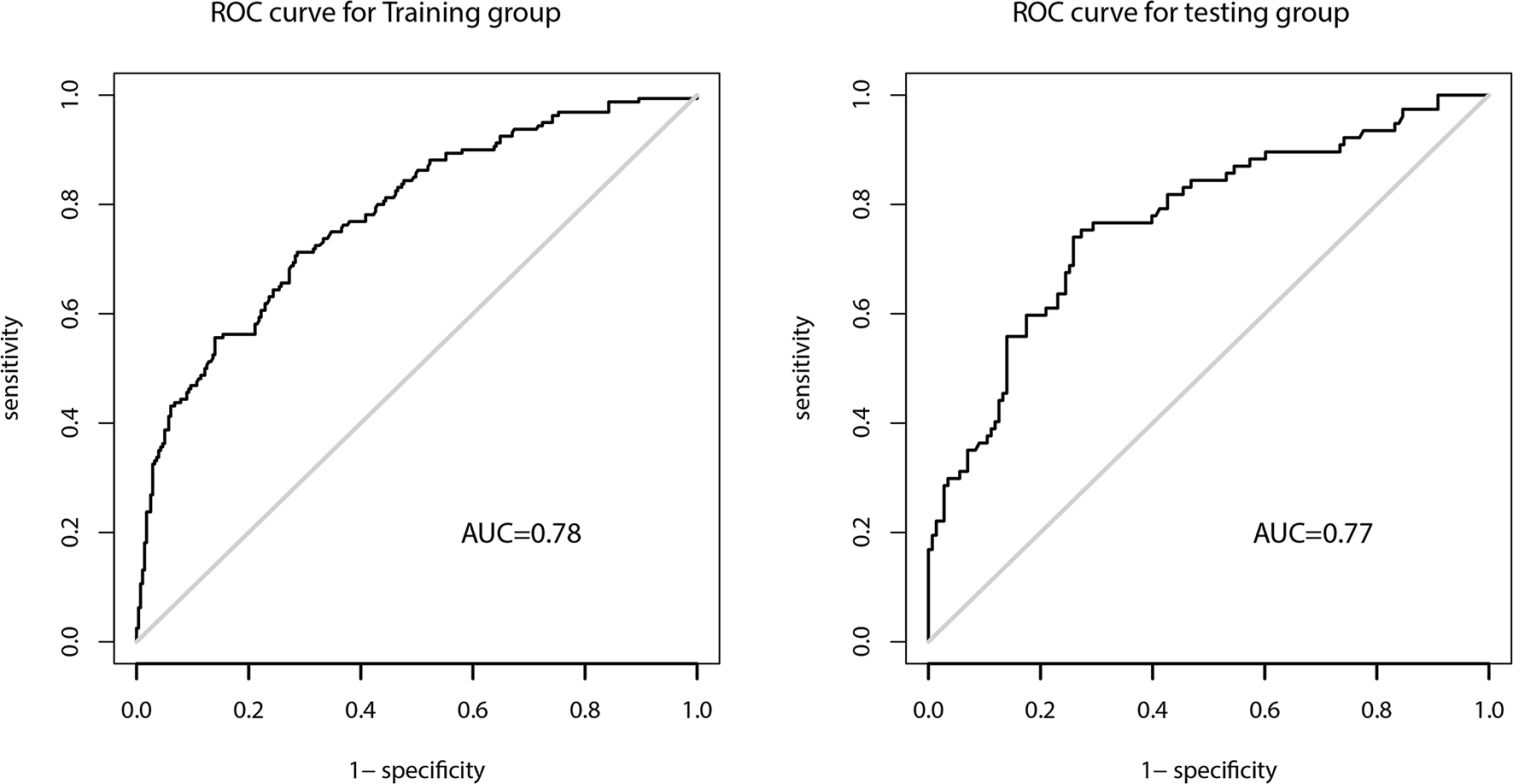
The ROC of prediction model in the development and validation cohorts.

**Figure 5:**
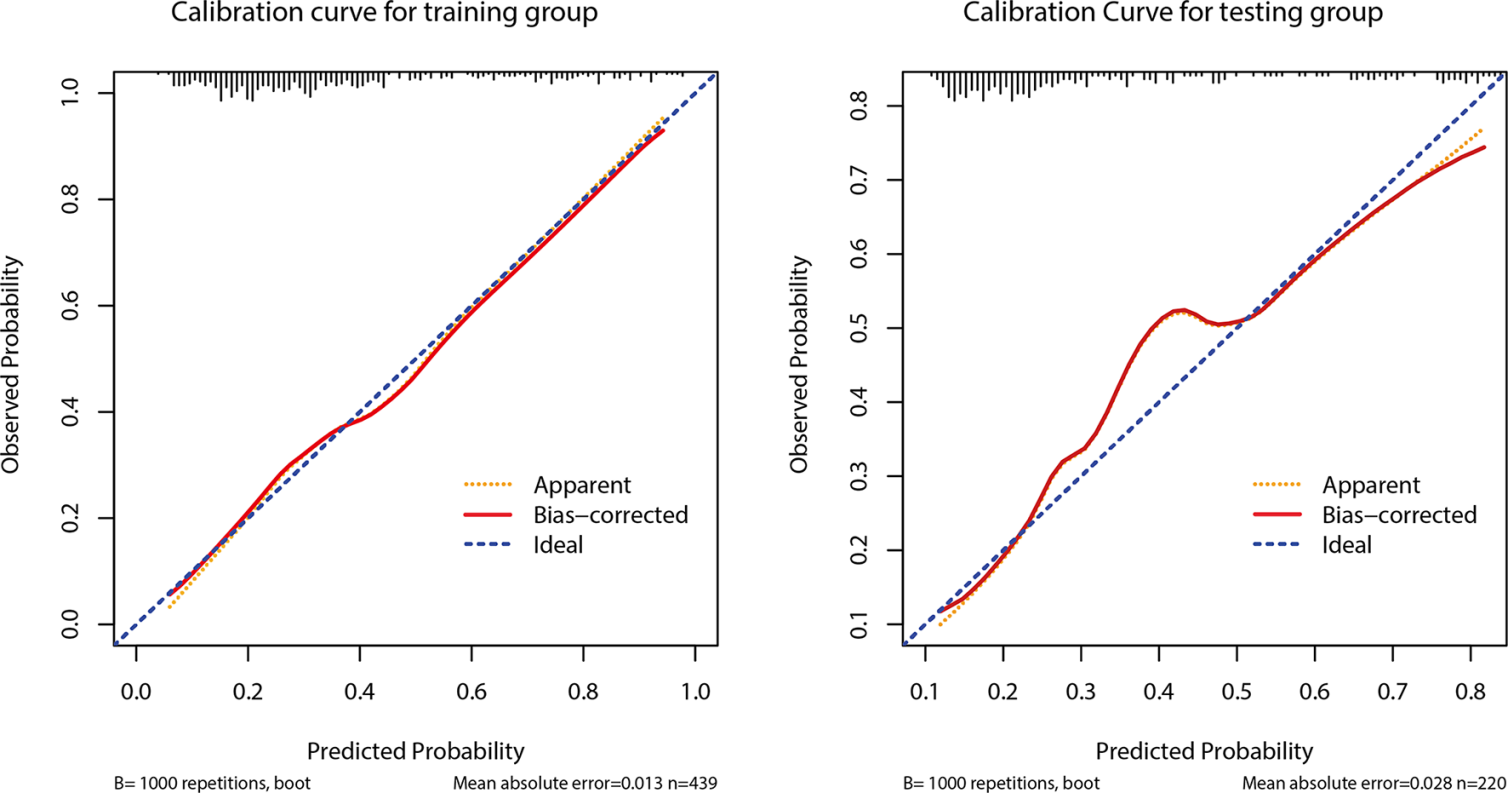
Calibration curves of the prediction model in the development and validation cohorts.

The AUC of the prediction model in the development set was 0.78 (95% CI: 0.735-0.825), and in the validation set was 0.77 (95% CI: 0.703-0.837) (**Figure 4**). **Figure 5** showed the calibration curve of CLNM predicted by the prediction model. The Hosmer–Lemeshow test of the predictive model in the development set showed no significant difference (*P* = 0.956), demonstrating that the model fitted well. The calibration curve shows that the predicted value is in good agreement with the actual value, and there is no deviation from the ideal model. The Hosmer-Lemeshow test of the predictive model in the validation set also did not show a significant difference (*P*=0.343). According to the calibration curve, when the prediction probability was between 22% and 50%, the development model slightly overestimated the prevalence (**Figure 5**).

### Decision Curve Analysis and Clinical Impact Curve

**Figure 6:**
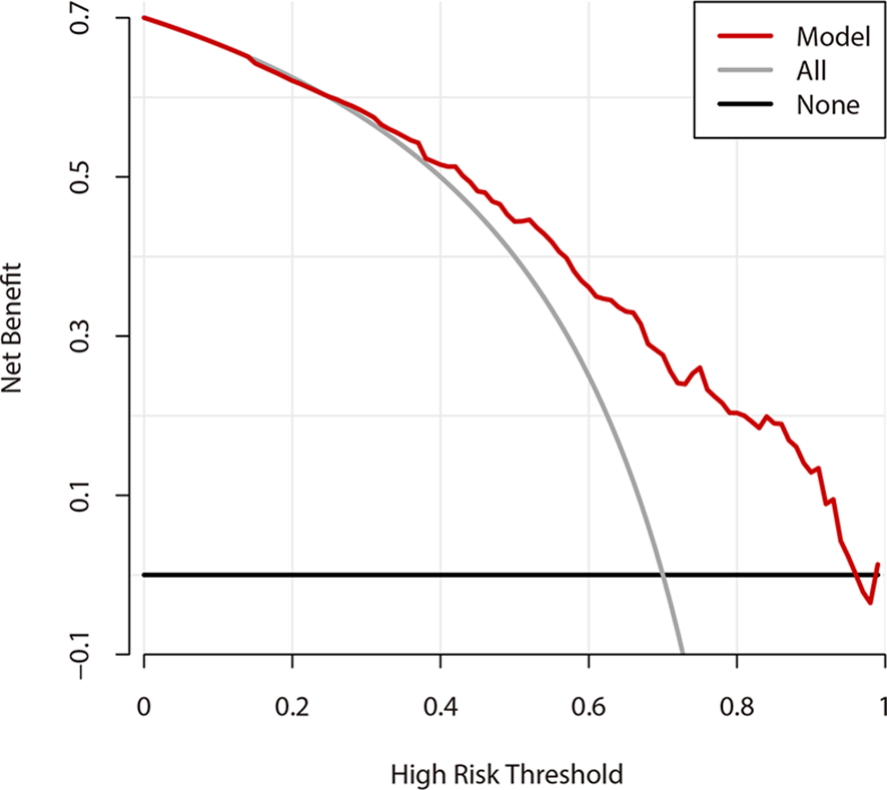
Decision curve analysis of prediction model.

**Figure 6** shows the DCA results of the prediction model. The abscissa represents the threshold probability, and the value on the ordinate represents the net benefits. The prediction model is represented with a red line. The gray line assumes that all patients have CLNM, while the black line indicates the hypothesis that all patients didn’t have CLNM. According to DCA, when the threshold probability was between 25-95%, the US prediction model can achieve better net benefits. Additionally, when the threshold probability was less than 25%, AS would be better.

**Figure 7:**
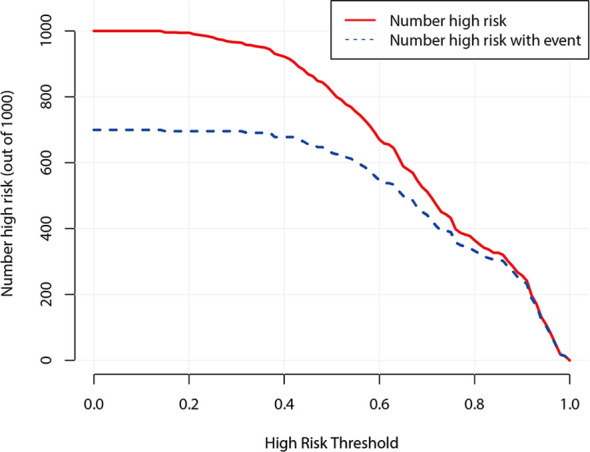
Clinical impact curve of prediction model.

**Figure 7** demonstrates the clinical impact curve of the predictive model. The X-axis represents the threshold probability, and the Y-axis is the number of high-risk cases (based on the prediction of 1,000 cases). The red curve (number high risk) indicates the number of cases predicted as positive (high risk) by the US predicting model at each threshold probability; the blue curve (number high risk with event) refers to the number of true positive cases under each threshold probability. The curve shows that the number of positive cases predicted by the model was close to the actual number of positive cases. As the risk threshold became higher, the number of cases predicted by model was closer to the actual number of cases.

### Web-Based Dynamic Calculator

**Figure 8:**
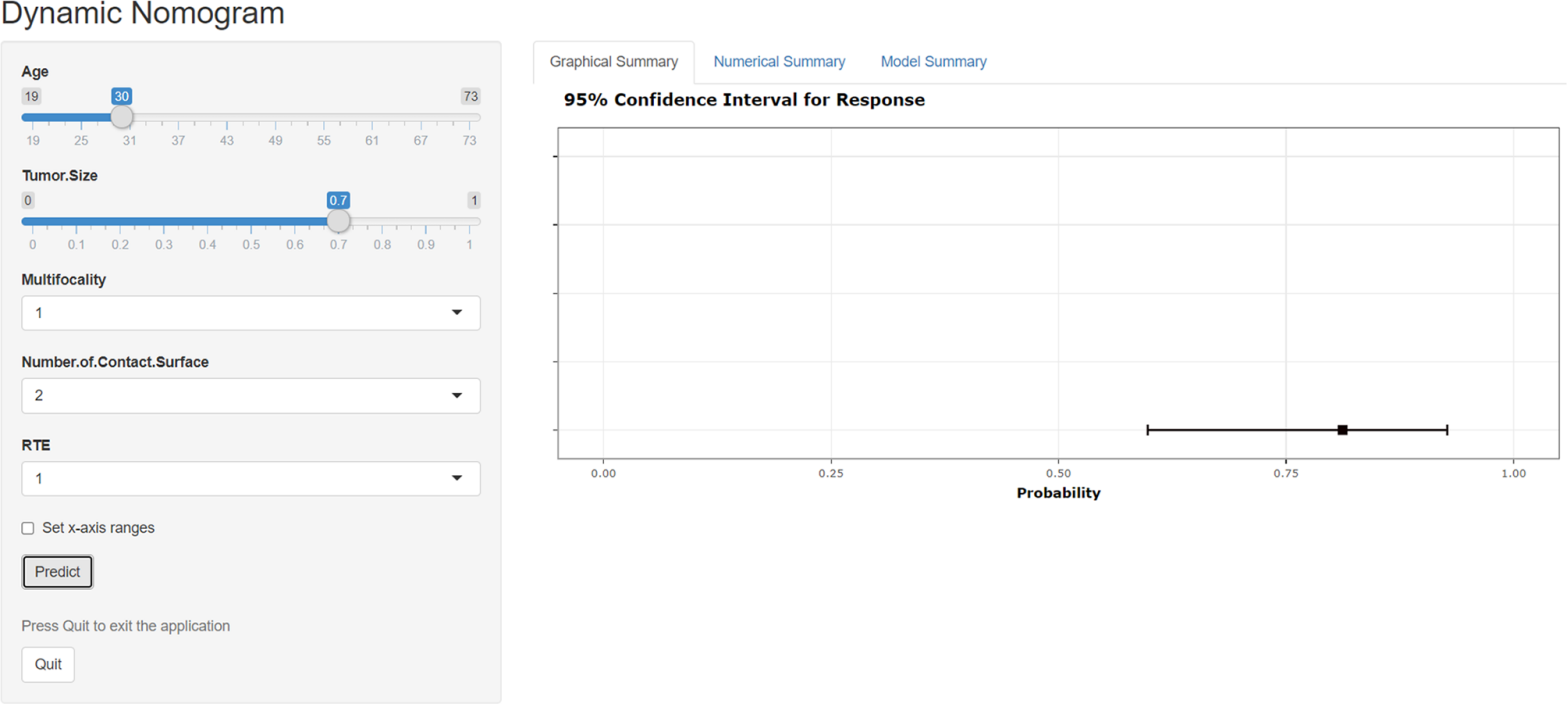
Screen shot of the dynamic web-based nomogram used to predict central lymph node metastasis in cN0 papillary thyroid microcarcinoma (https://predictclnminptc.shinyapps.io/DynNomapp3/).

Based on the results of DCA, a web-based dynamic calculator (https://predictclnminptc.shinyapps.io/DynNomapp3/) was generated to forecast the likelihood of CLNM in patients with cN0 PTMC. It is very convenient to input the risk prediction factors, such as patient’s age and US image features on the web page for real-time personalized prediction of patient’s CLNM probability. For example, the black line represents the probability (81.2%) and 95% CI (0.598-0.927) of CLNM in patients who are 30 years old, tumor diameter of 0.7 cm, multifocality, NCS ≥ 2, and ES of 3 (**Figure 8**).

## Discussion

The study showed the occurrence of CLNM in patients who had cN0 PTMC was 36% (237/659), while other studies showed that the occurrence of CLNM in patients who had cN0 PTMC confirmed by histopathology was 31-60.9% (2, 20). Most cN0-PTMCs are inert and have a good prognosis. Conservative treatment including AS may be safe (21). However, many healthcare providers still express various concerns regarding AS provision in real life, because several studies have found that patients with PTMC and CLNM were more prone to relapse and poor prognosis (22, 23). When clinical CLNM occurs during AS, patients will receive a wider range of treatment despite the low incidence, causing more complications (4). Additionally, the psychological factors of patients are also a key factor that influences the choice of clinicians. Therefore, it is essential to determine the risk probability of CLNM, which could help patients who have low risk of cN0 PTMC to choose AS with more confidence. Even if patients choose to undergo surgery, clinicians can decide whether to perform CCND according to risk probability. As far as we know, this study has established a dynamic nomogram based on the network for the first time, combining clinical and US risk predictors to forecast the probability of CLNM in cN0 PTMC patients. This nomogram could achieve a real visualization with better operability and practicality.

According to the multivariable regression analysis, our study found that young patients, maximum tumor diameter, multifocality, NCS, and RTE were independent risk factors that may predict CLNM. These predictors can be easily obtained before surgery. The model built in this study shows a high predictive value in the development and validation cohorts, and good consistency and recognition ability. Patients with more NCS and higher RTE scores may be, relatively, at higher risk of CLNM. Moreover, young patients with multifocality and larger tumor size should not be ignored, they are more likely to develop CLNM.

The threshold for tumor size and age reported in the literature vary considerably (1, 12–14, 23, 24). For example, the threshold for age was 40 (25), 43 (26), 45 (14), and 55 years (24). The threshold for tumor size was 0.5 mm (27), 0.6 mm (13), 0.7 mm (23, 26), and 0.75 cm (12). Considering these differences and the convenience of clinical practice, we selected tumor size and age as continuous variables to analyze and build a prediction model. In this study, CLNM risk increased with decrease of age, in agreement with other studies (14, 24–26). These studies have shown that younger age suggests worse prognosis; therefore, Zhang et al. (28) proposed that dynamic observation of the central lymph nodes may be an option for patients over 60 years of age. Controversial findings were also reported with respect to sex. Some studies (16, 29) showed, through multivariable regression analysis, that sex was significantly correlated with CLNM in PTMC patients, while others showed the opposite effect (14, 30). Our study got a higher CLNM rate significantly in males (71/152, 46.7%) than in females (166/507, 32.7%), *P* < 0.001. However, the multivariable analysis did not demonstrate a significant relationship between sex of cN0 PTMC patients and CLNM. The conclusion was consistent with the previous study (14).

As part of the characteristics of thyroid nodules, US elastography, especially the use of semi-quantitative method, has been of primary importance in the preoperative quantitative evaluation of thyroid malignancy (31). Eventhough there are some limitations in the diagnostic value of ultrasound elastography for large nodules and coarse calcified nodules. However, PTMC usually comprises small and solid nodules. Therefore, diagnosis of PTMC with elastography is not characterized by such limitations. A meta-analysis (32) reported that the pooled sensitivity and specificity of RTE in the diagnosis of thyroid malignant nodules were higher than that of shear-wave elastography (SWE) recently. The AUC of SWE and RTE were 0.842 and 0.885 individually. This meta-analysis uncovered that RTE had greater diagnostic value than SWE in distinguishing malignant from benign nodules (32). Furthermore, the ES of RTE was intensely associated with the malignant risk stratification of TI-RADS (33). Some researches applied SWE to forecast CLNM in PTC patients, the results demonstrated that SWE can improve the performance for preoperative lymph node staging (34, 35). Nevertheless, no literature has been reported so far regarding RTE predicting CLNM in patients who have cN0 PTMC. We found that there is a significant correlation between ES and CLNM. The probability of CLNM in cN0 PTMC patients growths with increased ES, and ES 4 showed the strongest predictive performance. The reason can be that extracellular matrix cross-linking takes a vital action in the biological aggressiveness of cancer cells and associates with tissue stiffening in tissue fibrosis (36, 37). Tumor progression is accompanied by cell proliferation and fibrosis, which may influence the hardness and invasiveness of the tumor, including the evolution of lymph node metastasis (38).

A recent study using univariate analysis showed that the US feature in recognizing capsule status was related to CLNM, and the sensitivity and specificity of US assessing capsule were 71.05% and 89.27%, respectively (39). A retrospective study revealed that negative predictive value for the extrathyroidal expansion (ETE) based on detailed US appraisal was 100%, which could reliably exclude microscopic ETE and reduce the proportion of avoidable total thyroidectomy from 57% to 31% (40). Therefore, preoperative evaluation of the capsule is valuable for the upcoming clinical strategy. This study analyzed the performance of US in evaluating NCS and predicting the risk probability of CLNM. As far as we know, no previous literature has studied US-based NCS of PTMC. US-based NCS incorporated the US evaluation of capsule infiltration to a large extent, and many studies have investigated the US evaluation of capsule infiltration. Hence, this study chose NCS as the observation index. Our research revealed that US-based NCS was an independent predictor of CLNM in cN0 PTMC patients. The possibility of CLNM increased with an increase of NCS.

One research suggested that multifocality and CLNM do not have any significant correlation (16). However, this study and other researches (9, 24, 26, 41) proved that multifocality was one of the high-risk factor for CLNM. Furthermore, other study has shown that pathological CLNM in PTMC patients was significantly correlated with US-reported CLNM, multifocality, and bilateral tumors (6). We found that multifocality and bilateral tumors were both significantly correlated with CLNM through univariate analysis. Moreover, the multivariable logistic regression analysis revealed that bilateral tumors were excluded from the prediction model, which may be due to multifocality incorporated bilateral tumors and US is more accurate in diagnosis of multifocal PTMC.

As a very practical, intuitive and innovative tool, the nomogram has been widely recognized (42–44). Although some previous studies have developed nomograms to forecast the likelihood of CLNM in PTMC patients, including clinical data and postoperative pathological features, most of these predictors were obtained after surgery, and did not include preoperative US features (24). Furthermore, some researchers have studied the association between the US findings of PTMC patients and the cervical lymph nodes, but no nomogram was established or validated (45, 46). To fill this gap, this study built a personalized and quantitative nomogram to forecast the CLNM risk probability of cN0 PTMC patients based on five preoperative independent predictors. The results fully prove that the nomogram in this study shows a favorable predictive ability for CLNM in cN0 PTMC patients. In addition, a web-based dynamic nomogram was constructed, which assisted in clinical management, and can provide a personalized and visual prediction for clinicians and patients at any time and place in the network.

Some limitations exist in our study. First of all, the nomogram was formed in a single-center study, which may be biased by the institutional diagnostic model. Therefore, it is necessary to conduct further prospective, multi-center, and larger sample size studies. Second, US is limited to small (less than 2 mm) lesions; thus, it is often unable to find all lesions, which may underestimate the role of multifocality and bilateral tumors in predicting CLNM (34). Finally, evaluation of some US features is subjective, and inter-observer variability may occur. Therefore, a third radiologist participated in the final decision to reduce inter-observer disagreement. Our retrospective study preliminarily explored the possibility of US features to predict CLNM in cN0 PTMC patients, and it is necessary to conduct prospective, multi-center, and larger sample size studies.

In conclusion, the web-based dynamic nomogram incorporating US and clinical features was able to forecast the risk of preoperative CLNM in cN0 PTMC patients, and has good predictive performance. As a new observational indicator, NCS can provide additional predictive information. The possibility of CLNM increased with an increase of NCS and ES.

## Data Availability Statement

The raw data supporting the conclusions of this article will be made available by the authors, without undue reservation.

## Ethics Statement

The studies involving human participants were reviewed and approved by The Research Ethics Committee of Guangdong Provincial People’s Hospital, Guangdong Academy of Medical Sciences. Written informed consent from the participants’ legal guardian/next of kin was not required to participate in this study in accordance with the national legislation and the institutional requirements.

## Author Contributions

CH, SC, and BZ designed this study. SS, MW, HZ, SW, and XA collected the data. CH, BZ, SS, and ZL analyzed the data. All authors contributed to the article and approved the submitted version.

## Funding

This work was supported by the Guangzhou Municipal Science and Technology Planning Project (CN) (202002030235, 202102020004), Medical Scientific Research Foundation of Guangdong Province (A2019080).

## Conflict of Interest

The authors declare that the research was conducted in the absence of any commercial or financial relationships that could be construed as a potential conflict of interest.

## Publisher’s Note

All claims expressed in this article are solely those of the authors and do not necessarily represent those of their affiliated organizations, or those of the publisher, the editors and the reviewers. Any product that may be evaluated in this article, or claim that may be made by its manufacturer, is not guaranteed or endorsed by the publisher.
